# Guiding Esthetic Crown Lengthening: A CBCT-Based Modified Classification of Altered Passive Eruption

**DOI:** 10.3390/dj14010067

**Published:** 2026-01-20

**Authors:** Kitichai Janaphan, Thanasak Rakmanee

**Affiliations:** 1 Center for Implant Dentistry and Periodontics, Faculty of Dentistry and Research Unit in Innovations in Periodontics, Oral Surgery and Advanced Technology in Implant Dentistry, Thammasat University, Patum Thani 12120, Thailand; kitichai@tu.ac.th; 2 Department of Restorative and Esthetic Dentistry, Faculty of Dentistry, Thammasat University, Patum Thani 12120, Thailand

**Keywords:** altered passive eruption, crown lengthening, excessive gingival display, classification, cone beam computed tomography

## Abstract

**Background:** Altered passive eruption (APE) is one of the etiological factors associated with excessive gingival display and is commonly treated with esthetic crown lengthening (ECL). However, existing classification systems provide limited guidance for selecting appropriate treatment approaches. **Objectives:** The aim of this study was to evaluate (1) the expected outcome of ECL in eliminating unattractive excessive gingival display (4 mm) based on digital smile assessment and (2) the distribution of teeth and patients according to the modified APE classification. **Methods:** Forty-two Thai patients with APE underwent clinical examination, digital smile assessment, intraoral scanning, and CBCT. Predicted gingival display (PGD) was calculated to assess the expected outcomes of ECL. The modified APE classification, incorporating CEJ–BC distance and buccal bone thickness, was analyzed at both the tooth and patient levels. **Results:** A total of 252 maxillary anterior teeth were assessed. Most patients (78.57%) presented with APE and hyperactive upper lip. The mean gingival display (GD) was 6.04 ± 1.76 mm, with GD ≥ 4 mm observed in 92.86% of patients. The mean PGD was 3.56 ± 1.71 mm, and ECL was predicted to reduce GD to < 4 mm in 66.67% of patients. Teeth were classified as Class I (28.97%), II (15.48%), III (41.27%), and IV (14.28%); only Types II (11.9%) and III (88.1%) occurred at the patient level. **Conclusions:** ECL performed at the CEJ level is predicted to eliminate excessive gingival display in approximately two-thirds of APE patients. The modified APE classification offers guidance for selecting surgical approaches, highlighting the necessity of open-flap procedures and the limited applicability of flapless approaches.

## 1. Introduction

Excessive gingival display, often described as a gummy smile, is defined as gingival display exceeding 2 mm during smiling [1]. A display greater than 4 mm is often considered unattractive, prompting many patients to seek treatment [2,3,4,5]. This condition can significantly affect self-esteem, perceived attractiveness, confidence, and overall quality of life [4,6,7], thereby justifying intervention. Excessive gingival display arises from multiple etiologic factors [8,9,10,11]. Altered passive eruption (APE) occurs when the gingiva fails to migrate apically during tooth eruption, resulting in a shortened clinical crown length (CCL) [12,13,14]. Hypermobile or hyperactive upper lip (HUL) is defined by lip mobility exceeding 8 mm between the rest and full smile positions [9]. Together, APE and hyperactive upper lip account for most cases of excessive gingival display cases [8,9]. Clinicians managing these cases are therefore likely to encounter and treat patients presenting with these conditions. APE is commonly managed with esthetic crown lengthening (ECL) [15], while hyperactive upper lip may be treated using lip repositioning surgery or botulinum toxin injections [10,14].

The primary goals of ECL are to increase clinical crown length, correct dentogingival disharmony, and reduce gingival display, thereby enhancing patient satisfaction with their smile [15,16,17]. In digital smile design and the fabrication of surgical guides, the cemento-enamel junction (CEJ) is frequently selected as a key anatomical landmark for determining the level of gingivectomy [18,19,20]. Recently, the concept of multifunctional anatomical prototypes was introduced [21], which also utilizes the anatomical CEJ—segmented using artificial intelligence (AI)—as a reference for both gingivectomy and osseous surgery in non-restorative cases. In this approach, soft tissue resection is limited to tissue coronal to the CEJ to avoid root exposure. From a patient perspective, a satisfactory outcome may include not only increased clinical crown length but also the elimination of gingival display ≥ 4 mm. However, the extent to which using the CEJ as a surgical reference in ECL achieves this esthetic goal remains unclear.

According to Coslet’s classification [13], APE is divided into Types I and II, each with subtypes A and B. This classification incorporates keratinized tissue width, mucogingival junction (MGJ) position, and the vertical distance from the bone crest (BC) to the CEJ, referred to as CEJ–BC. Type I is characterized by an excessive width of keratinized tissue with the MGJ positioned apical to the BC, whereas Type II presents with a normal width of keratinized tissue and the MGJ positioned at the level of the CEJ. Subtype A exhibits an adequate CEJ–BC distance, whereas subtype B presents with a coronally positioned BC in close proximity to the CEJ. Esthetic crown lengthening may be performed using either open-flap or flapless approaches, utilizing instruments such as scalpels, rotary burs, lasers, or piezosurgical devices [10,22,23,24]. A recent systematic review indicates that the flapless approach is less invasive, results in reduced postoperative discomfort, and facilitates faster healing [25]. However, this method may not be suitable for cases involving thick bone [14]. In such instances, open-flap surgery with osteoplasty is required to achieve proper flap adaptation, prevent bony ledge formation after osteotomy, and minimize coronal soft tissue rebound.

A recent treatment guideline for perio-restorative ECL has been proposed to facilitate both restorative and surgical planning [26]. However, a classification system to guide ECL in non-restorative cases—particularly one that informs the choice between conventional open-flap surgery and minimally invasive or flapless techniques, as well as overall procedural complexity—remains lacking. In response, the authors proposed a modified APE classification intended to provide more practical and comprehensive guidance for surgical planning in these scenarios.

Therefore, this study aimed to evaluate (1) the predicted outcome of ECL in eliminating unattractive gingival display, defined as greater than 4 mm, based on digital smile assessment, and (2) the distribution of teeth and patients according to the modified APE classification.

## 2. Materials and Methods

### 2.1. Study Design

This cross-sectional observational study involved the same patient cohort as reported in a previous study [8]. The study adhered to the Strengthening the Reporting of Observational Studies in Epidemiology (STROBE) guidelines [27]. Two calibrated examiners (YP and RT) conducted all measurements. Calibration was performed using 10 cases, achieving an intraclass correlation coefficient (ICC) of 0.802 (95% CI: 0.61–0.93).

### 2.2. Eligibility Criteria

The eligibility criteria have been described previously [8]. In brief, adult Thai patients presenting with gingival display greater than 2 mm during a full smile were initially screened. All patients diagnosed with APE, with or without concomitant etiologies such as hyperactive upper lip, were included in the analysis. APE was defined when the distance between the gingival margin and the CEJ exceeded 2 mm in at least two maxillary anterior teeth [28], while hyperactive upper lip was defined as upper lip mobility greater than 8 mm between the resting and full smile positions [9].

### 2.3. Clinical Examination and Digital Data Acquisition

Keratinized tissue width was measured in millimeters from the gingival zenith to the mucogingival junction (MGJ) using a Williams probe (Hu-Friedy, Chicago, IL, USA). Clinical photographs were captured using a digital single-lens reflex (DSLR) camera (Nikon D7000, Tokyo, Japan). Full-arch intraoral scans were acquired (3Shape A/S, Copenhagen, Denmark) and exported as standard tessellation language (STL) format. Cone-beam computed tomography (CBCT) images were acquired using the WhiteFox system (ACTEON Group, Olgiate Olona, Italy) with lip retractors (OptraGate, Ivoclar, Bangkok, Thailand), using parameters of 105 kVp, 9 mA, 9 s exposure, a voxel resolution of 0.2 mm^3^, and an 80 × 80 mm field of view (FOV), and were stored in Digital Imaging and Communications in Medicine (DICOM) format.

### 2.4. Digital Smile Assessment

**Figure 1:**
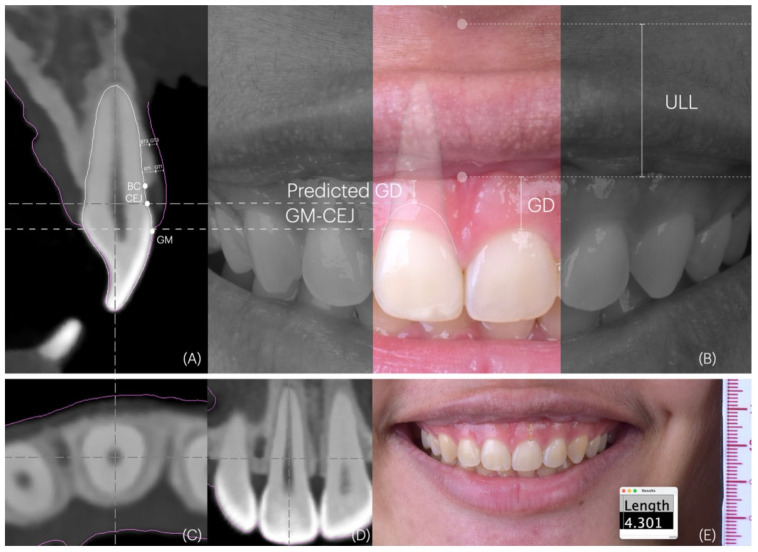
Digital smile assessment and measurement workflow. The pink outline represents the superimposition of STL and DICOM datasets using implant planning software. (**A**) Cross-sectional CBCT image showing key reference landmarks and measurements, including the gingival margin, cemento-enamel junction (CEJ), bone crest (BC), gingival thickness at 1 mm (GT1) and 3 mm (GT3) apical to the bone crest, and buccal bone thickness measured at 1 mm (BT1) and 3 mm (BT3) apical to the bone crest. (**B**) Clinical photograph obtained during a full smile showing upper lip length (ULL), gingival display (GD), and predicted GD, calculated by subtracting the GM–CEJ distance at the maxillary central incisors from the measured GD. The dotted line represents the anticipated excised gingiva and the predicted gingival level following gingivectomy. (**C**) Measurements performed at the mid-buccal aspect after standardizing each tooth orientation along its long axis. (**D**) image alignment protocol, including centering mesiodistally in the axial view, alignment along the root axis in tangential and cross-sectional views, and positioning through the mid-buccal crown. (**E**) Measurements of gingival display on standardized digital photographs using ImageJ software.

Gingival display was assessed at maxillary central incisors by measuring the mean vertical distance from the gingival margin to the upper lip during a full smile (Figure 1E). Upper lip length was defined as the vertical distance from the subnasale to the vermilion border of the upper lip, measured at rest and during a full smile (Figure 1B). Lip mobility was then calculated as the difference in upper lip length between these two positions. All measurements were performed on standardized digital photographs using image analysis software (ImageJ version 1.53t; NIH, Bethesda, MD, USA).

STL and DICOM files were superimposed using implant planning software (coDiagnostiX version 10.8; Dental Wings Inc., Montreal, QC, Canada). An artificial intelligence (AI)-assisted tool was used for registration and segmentation of tooth roots, with manual adjustments applied as needed. To ensure standardized and repeatable measurements within and across subjects, a consistent reference axis was established for each of the six maxillary anterior teeth. Measurements were performed at the mid-buccal aspect, following the long axis from the root apex to the incisal edge. Views were aligned as follows: centered mesiodistally in the axial view (Figure 1C), aligned the root axis in the tangential and cross-sectional views, and positioned through the mid-buccal crown (Figure 1D). This position was fixed and maintained for all subsequent measurements. In cross-sectional images, anatomical reference points including the gingival margin, CEJ, and bone crest (BC) were identified. Buccal bone thickness (BT) was assessed at 1 mm (BT_1_) and 3 mm (BT_3_) apical to the bone crest. Gingival thickness (GT) was measured at the level of the CEJ (GT-CEJ). Measurements were obtained by drawing perpendicular lines from the root surface to the corresponding hard- and soft-tissue outlines. Vertical distances from the gingival margin to the CEJ (GM–CEJ) and from the CEJ to the bone crest (CEJ–BC) were also recorded (Figure 1A).

Clinical crown length (CCL)was determined as the linear distance from the incisal edge to the gingival margin at the mid-buccal aspect, using the same reference described above. Anatomical crown length (ACL) was measured from the incisal edge to the CEJ at the same site. Crown width (CW) was defined as the maximum mesiodistal width of the tooth. The CW/CCL and CW/ACL ratios were then calculated.

### 2.5. Predicted Gingival Display (PGD)

PGD was derived by subtracting the GM–CEJ distance from the measured gingival display at the central incisors (Figure 1B). A successful outcome was defined as a PGD of less than 4 mm. PGD represents the estimated gingival display at the central incisors for each patient, assuming that ECL is performed successfully to the level of the CEJ.

### 2.6. The Modified APE Classification

A modified APE classification is proposed, consisting of two levels: tooth-level (Classes I–IV) and patient-level (Types I–III), as described below.

#### 2.6.1. Tooth-Level Classification Is Defined as Follows

**Figure 2:**
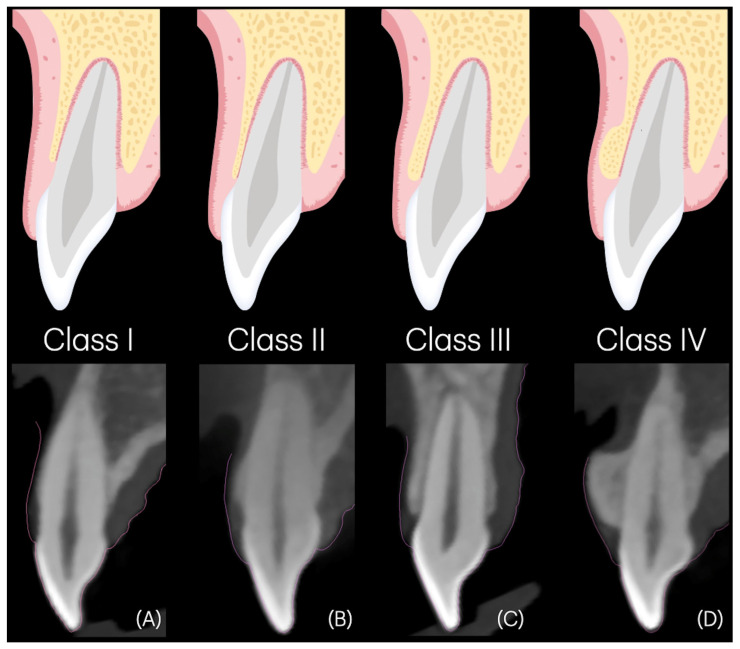
Modified APE classification based on CBCT measurements at the tooth level. (**A**) **Class I:** Distance from the cemento-enamel junction (CEJ) to the bone crest (BC) is ≥2 mm, regardless of buccal bone thickness (BT). (**B**) **Class II:** CEJ–BC distance < 2 mm, with BT ≤ 1 mm at both BT1 and BT3. (**C**) **Class III:** CEJ–BC distance < 2 mm, with BT > 1 mm but ≤2 mm at both BT1 and BT3. (**D**) **Class IV:** CEJ–BC distance < 2 mm, with BT > 2 mm or the presence of buccal exostosis.

Class I, a CEJ–BC distance ≥ 2 mm, regardless of buccal bone thickness (BT) (Figure 2A); Class II, a CEJ–BC distance < 2 mm with BT ≤ 1 mm at both BT1 and BT3 (Figure 2B); Class III, a CEJ–BC distance < 2 mm with BT > 1 mm but ≤2 mm at both BT1 and BT3 (Figure 2C); and Class IV, a CEJ–BC distance < 2 mm with BT > 2 mm or the presence of buccal exostosis (Figure 2D).

#### 2.6.2. Patient-Level Classification Is Defined as Follows

**Figure 3:**
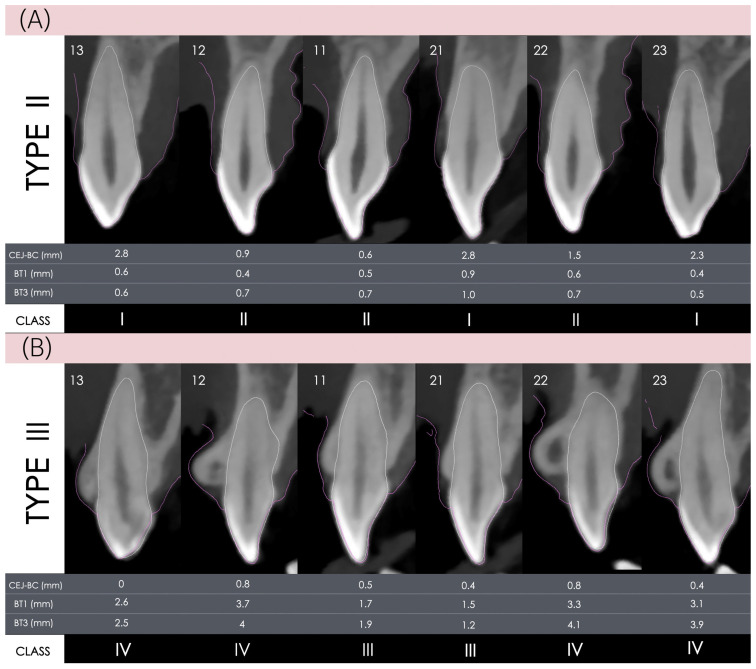
Representative case illustrating Type II and Type III according to the modified altered passive eruption classification. (**A**) **Type II** case, all anterior teeth are classified as either Class I or Class II. (**B**) **Type III** case, at least one anterior tooth is classified as Class III or Class IV. The pink outline represents the superimposition of STL and DICOM datasets using implant planning software. Tooth numbers 13–23 indicate the maxillary anterior teeth (canines, lateral and central incisors) according to the FDI tooth numbering system.

Type I, all anterior teeth are classified as Class I; Type II, all anterior teeth are classified as either Class I or Class II (Figure 3A); and Type III, at least one anterior tooth is classified as Class III or Class IV (Figure 3B).

### 2.7. Statistical Analysis

Descriptive analyses were used to summarize means, standard deviations (SDs), and frequencies. Independent *t*-tests were performed to compare gingival display, lip mobility, and gingival margin to CEJ distance between the APE-only and APE with hyperactive upper lip groups. The distribution of modified APE classifications across tooth types was analyzed using the chi-square test. Statistical analyses were conducted using SPSS software (version 28.0.1.1; IBM Corporation, Armonk, NY, USA), with the significance level set at 0.05.

## 3. Results

### 3.1. Demographic Information

Among the 88 patients initially assessed, two were excluded because of dental crowding associated with alveolar bone loss. Among the remaining 86 patients, only those diagnosed with APE, with or without hyperactive upper lip, were included, resulting in a final sample of 42 patients. The analysis included 252 maxillary anterior teeth, comprising 84 central incisors, 84 lateral incisors, and 84 canines. The mean age of participants was 27.0 ± 3.24 years, with 37 (88.10%) identifying as female and 5 (11.90%) as male. Of the 42 patients, 9 (21.43%) presented with APE only, and 33 (78.57%) presented with APE with hyperactive upper lip. Mean (SD) values for clinical crown length, anatomical crown length, and their respective ratios are presented in Table S1, whereas Table S2 summarizes mean (SD) values for KTW, GM–CEJ, CEJ–BC, BT1, BT3, and GT–CEJ.

### 3.2. Gingival Display and Predicted Gingival Display (PGD)

The overall mean (SD) gingival display was 6.04 (1.76) mm, and 39 patients (92.86%) exhibiting gingival display ≥ 4 mm. The mean gingival display was 5.03 (1.12) mm in the APE-only group and 6.32 (1.83) mm in the APE with hyperactive upper lip group (Table 1). A statistically significant difference in mean gingival display was observed between the groups (*p* = 0.016). Gingival display ≥ 4 mm was present in 8 of 9 patients (88.89%) in the APE-only group and in 31 of 33 patients (93.94%) in the APE with hyperactive upper lip group.

Based on the digital smile assessment, PGD was calculated for each patient (Table 1). The overall mean (SD) PGD was 3.56 (1.71) mm, and 14 patients (33.33%) predicted to have gingival display ≥ 4 mm. The mean PGD was 2.36 (1.38) mm in the APE-only group and 3.89 (1.64) mm in the APE with hyperactive upper lip group. A statistically significant difference in mean PGD was observed between the groups (*p* = 0.015). PGD ≥ 4 mm was predicted in 1 of 9 patients (11.11%) in the APE-only group and in 13 of 33 patients (39.39%) in the APE with hyperactive upper lip group. Therefore, the overall predicted success rate for eliminating gingival display ≥ 4 mm was 66.67% (28 patients), with 8 of 9 patients (88.89%) in the APE-only group and 20 of 33 patients (60.61%) in the APE with hyperactive upper lip group.

In addition, the mean upper lip mobility and GM–CEJ distance at the maxillary central incisors for each group are summarized in Table 1. Lip mobility was significantly greater in the APE with hyperactive upper lip group (*p* < 0.001), whereas no significant difference in GM–CEJ distance was observed between groups (*p* = 0.252).

### 3.3. Distribution of Teeth and Gummy Smile Patients According to the Modified APE Classification

Teeth were classified according to the modified APE system as follows: Class I (73 teeth, 28.97%), Class II (39 teeth, 15.48%), Class III (104 teeth, 41.27%), and Class IV (36 teeth, 14.28%) (Table 2). At the patient level, no individuals were identified as Type I, while Type II and Type III were observed in 5 (11.90%) and 37 (88.10%) patients, respectively. When analyzed by tooth (Table 3), a chi-square test demonstrated a statistically significant difference in APE class distribution (*p* = 0.001), indicating that the classes were not equally distributed. Notably, Class IV occurred more frequently in canines.

## 4. Discussion

This study evaluated the predicted outcome of ECL in eliminating gingival display greater than 4 mm and assessed the distribution of cases according to the modified APE classification. Among the 42 patients with APE, 39 (92.86%) exhibited gingival display greater than 4 mm. Smile assessment showed that ECL performed to the level of the CEJ was predicted to successfully eliminate gingival display ≥ 4 mm in 28 patients (66.67%). At the tooth level, Classes I, II, III, and IV accounted for 28.97%, 15.48%, 41.27%, and 14.28% of teeth, respectively. At the patient level, no cases were classified as Type I, whereas Types II and III were observed in 10.87% and 89.13% of patients, respectively, indicating that most cases required an open-flap approach.

Patients with both APE and hyperactive upper lip presented with significantly greater gingival display, likely due to increased lip mobility. Consequently, the predicted success rate in this group was lower, approximately 60%, compared with 88.89% in the APE-only group. As gingival display greater than 4 mm is generally considered an esthetically unacceptable threshold by both clinicians and laypersons [2,3,4,5,29], reduction below this level is critical for patient satisfaction and improvement in oral health–related quality of life (OHRQoL) [6]. In this study, PGD was estimated by subtracting the GM-CEJ distance from baseline gingival display. The mean GM–CEJ distance at the central incisors was approximately 2.5 mm, which may serve as a clinical reference for predicting achievable gingival display reduction following ECL.

Approximately one-third of patients were predicted to exhibit persistent gingival display greater than 4 mm following ECL. In such cases, particularly those with severe excessive gingival display greater than 6 mm, adjunctive treatments such as lip repositioning surgery or botulinum toxin injections may be necessary [10,30,31]. Lip repositioning has been reported to reduce gingival display by approximately 3 mm [32], although relapse rates of 25% within 12 months have also been reported [32]. Botulinum toxin can temporarily reduce hyperactive lip elevation, achieving an average gingival display reduction of 3.4 mm within two weeks; however, the effect typically diminishes after 24 weeks, necessitating repeat treatments [30,33]. Therefore, in patients with multifactorial etiology, ECL should be performed first to correct dentogingival disharmony, followed by re-evaluation for residual gingival display and additional interventions if required [34]. These approaches, however, may not apply to gummy smile cases of skeletal origin, such as vertical maxillary excess, or to soft tissue abnormalities, such as a short upper lip [35].

ECL outcomes are also influenced by anatomical and esthetic constraints, particularly the CEJ position, which prevents root exposure, and the target crown width-to-length (CW/CL) ratio [36,37,38]. Tooth proportion plays a key role in smile esthetics, with a CW/CL ratio of 0.75–0.80 generally considered ideal [39]. Ethnic differences have been reported, as Asian populations typically exhibit shorter and broader teeth than Caucasians [40,41]. In this study, patients with APE presented with short, square central incisors, with a mean crown length of 9.12 mm and a CW/CL ratio of 0.95. The anatomical crown length (ACL) measured 11.58 mm, which is consistent with previous Asian data [40]. Performing a gingivectomy to the CEJ level, or exposing the anatomical crown, may therefore contribute to correcting the CW/CL ratio to approximately 0.75, thereby aligning with the esthetic standard. These findings suggest that the CEJ represents a key anatomical landmark for determining the surgical position of gingivectomy in non-restorative cases, consistent with previous literature [16,18,42,43].

The original classification of altered passive eruption (APE) proposed by Coslet et al. (1977) remains widely used, dividing cases into Types I and II based on keratinized tissue width and mucogingival junction position, and into subtypes A and B according to the distance between the CEJ–BC [13]. However, this classification was derived solely from clinical observations. A recent CBCT-based study identified APE in 55% of patients and 35.5% of teeth, with most classified as Type I and equal proportions of subtypes A and B [8].

A subsequent modification incorporated gingival thickness as a parameter to guide surgical blade angulation and distinguished between APE alone and APE associated with altered active eruption [44]. While the original system aids in determining flap design, such as gingivectomy versus an apically positioned flap, and the necessity of osteotomy, it provides limited guidance for selecting the surgical approach, open-flap versus flapless, or estimating procedural complexity. CBCT imaging and digital superimposition enable a more comprehensive, patient- and site-specific evaluation of the dentogingival complex, supporting further refinement of the original APE classification [45,46].

This study proposed a modified APE classification derived from clinical and radiographic findings in the anterior maxilla. In this region, the mean keratinized tissue width was 6–7 mm, and nearly all APE cases were classified as Type I according to Coslet et al. [13]. Consequently, Type II, which is defined by keratinized tissue width variation, was excluded. The CEJ–BC distance, a critical determinant for performing ostectomy to restore the supracrestal tissue attachment dimension and prevent soft tissue rebound [12,14], was incorporated using a 2 mm threshold, consistent with previous studies on non-APE teeth [8,16]. Bone thickness, another key factor affecting the surgical approach, determines the feasibility of flapless versus open-flap procedures and the extent of osteoplasty required [25]. Thick bone morphotypes are common in APE cases [36], and 15% of teeth showing bone thickness more than 2 mm or buccal exostoses [47]. Incorporating bone thickness improves surgical planning and helps anticipate procedural complexity [23,24,25].

Approximately 30% of teeth were classified as Class I, for which gingivectomy alone is sufficient due to a CEJ–BC distance of ≥ 2 mm. Teeth classified as Class II, defined by a CEJ–BC distance < 2 mm and bone thickness ≤ 1 mm, require ostectomy to restore the 2 mm biologic space and may be treated with a flapless approach. However, this condition was observed in only 15.48% of teeth, suggesting limited applicability of minimally invasive flapless ECL [22,23,24,25].

Class III and Class IV were characterized by a CEJ–BC distance < 2 mm and thick bone morphotype. In Class III, bone thickness ranges from 1–2 mm, while in Class IV it exceeds 2 mm or involves buccal exostoses. These categories accounted for 41.27% and 14.28% of teeth, respectively. Class III was the most prevalent, consistent with reports that Type IB APE often requires osteoplasty for improved flap adaptation [14,48]. Open-flap ECL with ostectomy and osteoplasty is therefore recommended [10,14,49]. Class IV teeth may require extensive exostosis removal, which increases surgical complexity but may contribute to reducing lip mobility [50,51].

The modified classification incorporates both tooth-level (Classes I–IV) and patient-level (Types I–III) categories. This distinction reflects that, while APE may not involve all anterior teeth [8,28], ECL is usually performed on all teeth visible in the smile to achieve esthetic harmony. Type I represents straightforward cases suitable for gingivectomy alone, although none were observed in the present study. Type II includes cases requiring osteotomy with a thin buccal bone (<1 mm), in which flapless ECL may be feasible, minimizing postoperative discomfort and recovery time but necessitating specialized instruments such as lasers or piezoelectric devices [24,25,52]. Type III encompasses cases requiring both osteotomy and osteoplasty, thereby necessitating an open-flap approach.

This study is not without limitations. PGD is an estimate derived from calculated parameters and may not precisely reflect the actual gingival margin position following ECL, as soft tissue rebound of approximately 0.5 mm is commonly observed [16,36]. Moreover, various factors may influence tissue rebound [48,49,53,54]. The assessment of gingival display in this study was based on the mean value at the central incisors. However, posterior gingival exposure has been reported to exceed anterior exposure, with greater lip mobility observed in the canine region compared with the central incisors [33,55]. This modified classification requires the use of CBCT for evaluation, which may increase treatment costs and radiation exposure, thereby limiting its practical applicability [56,57]. Lastly, future clinical studies with larger sample sizes are warranted to validate the reliability and applicability of PGD and the proposed classification across different populations [58,59].

## 5. Conclusions

Despite the study limitations, ECL successfully eliminated gingival display ≥ 4 mm in approximately two-thirds of patients with APE. The modified APE classification indicated that gingivectomy alone (Class I) was applicable to approximately 30% of maxillary anterior teeth. Flapless ECL (Class II) was suitable for only about 15% of teeth. This suggests limited applicability, with most patients requiring open-flap ECL involving osteotomy and osteoplasty.

## Figures and Tables

**Table 1:** Comparison of gingival display (GD), Lip mobility, and Gingival margin to CEJ (GM–CEJ) distance and predicted gingival display (PGD) between APE-only and APE with hyperactive upper lip groups.

Etiology	N (Frequency %)	GD Mean (SD)	GD ≥ 4 mm N (Frequency%)	Lip Mobility Mean (SD)	GM-CEJ at Central Incisors Mean (SD)	Predicted		
						GD Mean (SD)	GD ≥ 4 mm N (Frequency%)	Success ** N (Frequency%)
**APE-only**	9 (21.43%)	5.03 (1.12)	8 (88.89%)	7.29 (0.72)	2.69 (0.83)	2.36 (1.38)	1 (11.11%)	8 (88.89%)
**APE + HUL**	33 (78.57%)	6.32 (1.83)	31 (93.94%)	10.65 (1.89)	2.44 (0.49)	3.89 (1.64)	13 (39.39%)	20 (60.61%)
***p*-value ^a^**	NA	0.016 *	NA	<0.001 *	0.252	0.015 *	NA	NA
**Overall** **(Total)**	42 (100%)	6.04 (1.76)	39 (92.86%)	9.93 (2.19)	2.46 (0.64)	3.56 (1.71)	14 (33.33%)	28 (66.67%)

N = Number of Subjects; GD = Gingival display; NA = not appliable; APE = Altered passive eruption; HUL = Hyperactive upper lip; ^a^ Independent *t*-tests; * Statistical significance, *p* < 0.05; ** Success was defined as a PGD of less than 4 mm.

**Table 2:** Modified APE Classification: Diagnostic Criteria, Distribution, Recommended Treatment, and Procedural Complexity at the Tooth and Patient Levels.

Modified APE Classification				
Classification	Diagnostic Criteria	Number (N) of Teeth or Patients (Frequency%)	Recommended Treatment	Procedural Complexity *
**Tooth-level (N = 252)**				
**Class I**	CEJ–BC distance ≥ 2 mm, regardless of buccal bone thickness (BT)	73 (28.97%)	Gingivectomy only	**+**
**Class II**	CEJ–BC distance < 2 mm, with BT ≤ 1 mm at both BT1 and BT3	39 (15.48%)	Gingivectomy + Flapless surgical crown lengthening	**++**
**Class III**	CEJ–BC distance < 2 mm, with BT > 1 mm but ≤2 mm at both BT1 and BT3	104 (41.27%)	Gingivectomy + Open-flap surgical crown lengthening (Ostectomy + minor osteoplasty)	**+++**
**Class IV**	CEJ–BC distance < 2 mm, with BT > 2 mm or the presence of buccal exostosis	36 (14.28%)	Gingivectomy + Open-flap surgical crown lengthening (Ostectomy + extensive osteoplasty)	**++++**
**Patient-level (N = 42)**				
**Type I**	All anterior teeth are classified as Class I.	0	Gingivectomy case	**+**
**Type II**	All anterior teeth are classified as either Class I or Class II.	5 (11.90%)	Flapless case	**++**
**Type III**	At least one anterior tooth is classified as Class III or Class IV.	37 (88.10%)	Open-flap case	**+++**

* Procedural complexity was categorized qualitatively, in ascending order, from (+) to (++++) based on the extent of surgical intervention required. This includes gingivectomy alone (+), flapless surgical crown lengthening with limited ostectomy (++), open-flap surgical crown lengthening with ostectomy and minor osteoplasty (+++), and open-flap surgery with extensive osteoplasty or management of buccal exostosis (++++). At the patient level, procedural complexity was determined by the highest tooth-level class present among the anterior teeth, reflecting the overall surgical demand of the case.

**Table 3:** Distribution of the modified APE classification classes by tooth type.

Class	Tooth Type		
	Central Incisors N (Frequency %)	Lateral Incisors N (Frequency%)	Canine N (Frequency%)
I	21 (25.00%)	27 (32.14%)	25 (29.76%)
II	15 (17.86%)	16 (19.05%)	8 (9.52%)
III	44 (52.38%)	31 (36.90%)	29 (34.52%)
IV	4 (4.76%)	10 (11.90%)	22 (26.19%)

N = Number of teeth.

## Data Availability

The original contributions presented in this study are included in the article/Supplementary Material. Further inquiries can be directed to the corresponding author.

## Supplementary Materials

The following supporting information can be downloaded at: https://www.mdpi.com/article/10.3390/dj14010067/s1, Table S1. Mean (SD) in mm of clinical crown length (CCL) and anatomic crown length (ACL), according to tooth type. CCL (clinical crown length); ACL (anatomical crown length). Table S2. Mean (SD) in mm of parameters, categorized by tooth type. KTW, keratinized tissue width; GM-CEJ, distance from the gingival margin to the cemento-enamel junction (CEJ); CEJ-BC, distance from the CEJ to the bone crest (BC); BT1 and BT3, buccal bone thickness measured at 1 mm and 3 mm apical to the bone crest, respectively; GT-CEJ, gingival thickness at the level of the CEJ.

## Abbreviations

The following abbreviations are used in this manuscript:

ACL	Anatomical crown length
AI	Artificial intelligence
APE	Altered passive eruption
BC	Bone crest
BT	Bone thickness
CBCT	Cone-beam computed tomography
CCL	Clinical crown length
CEJ–BC	Distance from the CEJ to bone crest
CEJ	Cemento-enamel junction
CW	Crown width
CW/ACL	Crown width/Anatomical crown length ratio
CW/CCL	Crown width/Clinical crown length ratio
CW/CL	Crown width-to-length ratio
DICOM	Digital Imaging and Communications in Medicine
ECL	Esthetic crown lengthening
GD	Gingival display
GM	Gingival margin
GM–CEJ	Distance from gingival margin to cemento-enamel junction
GT	Gingival thickness
GT–CEJ	gingival thickness measured at cemento-enamel junction
HUL	Hypermobile upper lip
ICC	Intraclass correlation coefficient
KTW	Keratinized tissue width
MGJ	Mucogingival junction
OHRQoL	Oral health–related quality of life
PGD	Predicted gingival display
SDs	Standard deviations
STROBE	Strengthening the Reporting of Observational Studies in Epidemiology
STL	Standard tessellation language
