# Supplementary material for: Guiding Esthetic Crown Lengthening: A CBCT-Based Modified Classification of Altered Passive Eruption
Source: Dent J (Basel). 2026 Jan 20;14(1):67. doi: 10.3390/dj14010067 (PMC12839652; doi:10.3390/dj14010067)
Supplement: Supplementary file 1 [file dentistry-14-00067-s001.zip › dentistry-4003910-supplementary.pdf]

Table S1. (Supplement) Mean (SD) in mm of clinical crown length (CCL) and anatomic crown length (ACL), according to tooth type.

	Crown Width	Crown Length	Width/Length Ratio
		Central incisors	
CCL	8.60 (0.56)	9.12 (1.03)	0.95 (0.11)
ACL		11.58 (0.89)	0.75 (0.07)
		Lateral incisors	
CCL	7.03 (0.52)	7.66 (0.91)	0.93 (0.11)
ACL		10.02 (0.82)	0.70 (0.07)
		Canines	
CCL	7.98 (0.55)	8.35 (0.80)	0.96 (0.09)
ACL		10.29 (0.79)	0.78 (0.07)

CCL (clinical crown length); ACL (anatomical crown length).

Table S2. (Supplement) Mean (SD) in mm of parameters, categorized by tooth type.

Parameters	Central Incisors N = 84	Lateral Incisors N = 84	Canines N = 84	All N = 252
KTW	6.58 (1.01)	6.64 (1.11)	6.12 (1.21)	6.45 (1.14)
GM-CEJ	2.46 (0.64)	2.36 (0.55)	1.94 (0.72)	2.25 (0.68)
CEJ-BC	1.63 (0.61)	1.76 (0.55)	1.75 (0.78)	1.71 (0.66)
BT1	1.24 (0.42)	1.24 (0.5)	1.57 (0.71)	1.35 (0.58)
BT3	1.23 (0.49)	1.20 (0.66)	1.62 (0.88)	1.35 (0.72)
GT-CEJ	1.39 (0.53)	1.29 (0.49)	1.32 (0.53)	1.33 (0.52)

KTW, keratinized tissue width; GM-CEJ, distance from the gingival margin to the cemento-enamel junction (CEJ); CEJ-BC, distance from the CEJ to the bone crest (BC); BT1 and BT3, buccal bone thickness measured at 1 mm and 3 mm apical to the bone crest, respectively; GT-CEJ, gingival thickness at the level of the CEJ.
